# Exfoliated Molybdenum Disulfide Nanosheet Networks as Sensing Materials for Nitrogen Dioxide Detection

**DOI:** 10.1021/acsanm.4c05066

**Published:** 2025-01-24

**Authors:** Kusuma Urs, Tian Carey, Leonidas Tsetseris, Shixin Liu, Kevin Synnatschke, Zdeněk Sofer, Jonathan N. Coleman, John Charles Wenger, Subhajit Biswas, Justin D. Holmes

**Affiliations:** † School of Chemistry, University College Cork, Cork T12 YN60, Ireland; ‡ AMBER Centre, Environmental Research Institute, University College Cork, Cork T23 XE10, Ireland; § School of Physics, Trinity College Dublin, Dublin 2, Ireland; ∥ Centre for Research on Adaptive Nanostructures and Nanodevices (CRANN) and Advanced Materials Bio-Engineering Research Centre (AMBER), Trinity College Dublin, Dublin 2, Ireland; ⊥ Department of Physics, School of Applied Mathematical and Physical Sciences, National Technical University of Athens, Athens 15780, Greece; # Department of Inorganic Chemistry, University of Chemistry and Technology Prague, Technická 5, Prague 166 28, Czech Republic

**Keywords:** MoS_2_, PET substrate, electrochemical exfoliation, NO_2_ sensing, UV light and DFT

## Abstract

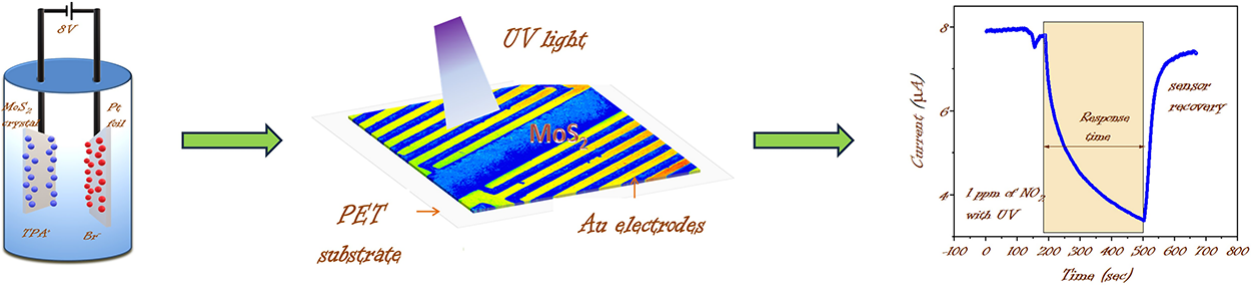

Nitrogen dioxide (NO_2_) is a gaseous air pollutant linked to respiratory and cardiovascular diseases and environmental problems such as acid rain and tropospheric ozone formation. Reference instruments for measuring NO_2_ are expensive, highlighting the need to develop low cost sensor technologies for wider scale monitoring of this critical pollutant. Here, we report the development of a scalable sensor using electrochemically exfoliated 2D molybdenum disulfide (MoS_2_) networks. The sensor can detect a wide range of NO_2_ concentrations at room temperature, with an experimental limit of detection (LOD) as low as 150 ppb and a theoretical LOD of 1.9 parts per quadrillion (ppq) in dry air. The sensor exhibited approximately 90% response to 1 ppm of NO_2_ within 10 min of exposure. UV irradiation significantly enhanced the sensor’s recovery time, reducing it from 20 min to less than 2 min. Evaluation of the sensor within a large (∼6.5 m^3^) atmospheric simulation chamber yielded a similar response and recovery time performance, opening the opportunity for further tests in the chamber under various conditions. Finally, using Density Functional Theory (DFT) calculations, we identified key atomic-scale structures and processes highlighting the importance of electrochemically exfoliated sulfur-deficient MoS_2_ for sensitive room temperature NO_2_ detection.

## Introduction

Poor air quality can profoundly impact health, ecosystems and overall quality of life. Therefore, it is crucial to maintain air quality for the well-being of both humans and the environment and economic prosperity. However, the emission of toxic gases from sources such as industry, vehicles and indoor activities primarily contributes to environmental pollution and reduces air quality. Nitrogen dioxide (NO_2_) is one of the hazardous gases that significantly impacts the health of living organisms.^1^ NO_2_ is released into the air through fuel combustion, industrial emissions, gas cooking and other sources.^2^ In addition to being a primary pollutant, NO_2_ readily undergoes photolysis in the atmosphere to form ground level ozone and reacts with the hydroxyl radical to produce nitric acid, a key component of acid rain.^3^ As an oxidizing gas, NO_2_ poses a serious threat to human health, causing short-term symptoms like breathing difficulties and long-term respiratory issues upon prolonged exposure.^4^ To address these concerns, regulatory agencies like the Environmental Protection Agency (EPA) and the European Commission have established exposure limits for NO_2_ and a range of other air pollutants.^5^

The harmful effects of NO_2_ emissions on human health and the environment underscore the importance of monitoring NO_2_ at ambient concentrations, typically 5–100 ppb, but can reach several hundred ppb in very polluted areas. Reference methods for measuring NO_2_ require expensive instrumentation, highlighting the need to develop low-cost sensor technologies for wider scale monitoring of this important pollutant. While significant advancements have been made in gas sensor technology over the past two decades,^6,7^ challenges remain related to real-time monitoring, room temperature operation, sensitivity and selectivity. To address these limitations, researchers have generally focused on solid-state sensors, which offer advantages such as affordability, high sensitivity and ease of integration into devices.^8^ Although commercial NO_2_ sensors primarily rely on electrochemical cells,^9^ they face limitations, including limited shelf life and a narrow range of operation temperatures (less than 50 °C). Chemiresistive sensors, which use materials such as metal oxides, conductive polymers and carbon-based substance^10^ are widely used. Metal oxides are particularly popular due to their cost-effectiveness and detection capabilities, but they require high operating temperatures and have limited selectivity. Conversely, conductive polymers degrade upon interaction with gases under certain environmental conditions, making them unsuitable for many applications,^11^ especially outdoor use in humid conditions. Therefore, there is a need to develop portable NO_2_ sensors that can operate at room temperature, consume low power and exhibit high sensitivity and selectivity for practical applications.

Transition metal dichalcogenides (TMDs) have emerged as promising materials for gas sensing applications^12^ due to their large surface area and sensitivity to the surrounding chemical environment.^13^ Despite extensive research on chemically synthesized and mechanically exfoliated molybdenum disulfide (MoS_2_) for gas detection, challenges such as recovery and selectivity persist, particularly when operating at room temperature.^14^ MoS_2_ sensors fabricated using chemical vapor deposition (CVD) techniques demonstrate high sensitivity with a low detection limit and shorter response times; however, they often suffer from inconsistent film quality. Similarly, sensors based on MoS_2_ synthesized via wet chemical methods exhibit longer response times and variable performance than those using liquid-phase exfoliation (LPE) methods.^12,15,16^ UV irradiation has proven to be a valuable enhancement for these sensors, as it generates electron–hole pairs that significantly improve sensitivity. UV light accelerates the charge exchange between the sensor surface and NO_2_ molecules while facilitating the desorption of physisorbed molecules, thereby improving both detection efficiency and recovery speed.

Most NO_2_ sensors reported in the literature are based on Si/SiO_2_ substrates, which is impractical for integration in wearable technology.^8^ This study aims to assess the response of sensors based on 2D MoS_2_ networks on flexible polyethylene terephthalate (PET) substrates to achieve heightened sensitivity across a broad detection range at room temperature, in the presence of other gases. Here, we present an electrochemical synthesis of MoS_2_ flakes using printing based on a Langmuir–Schaefer type approach^17^ to deposit thin films of MoS_2_ nanosheets on flexible and transparent PET substrates. PET substrates offer several advantages, including high mechanical flexibility, low cost and transparency. Their flexibility makes them particularly well-suited for wearable applications like smart watches and clothing, enabling real-time air quality monitoring. The sensitivity, selectivity and response of the electrochemically exfoliated MoS_2_ ink to the detection of NO_2_ in air has been explored at room temperature. Also, it provides valuable information for an individual to assess their environment for the air quality in various domains.

Furthermore, the sensor devices were tested in a large volume (6.5 m^3^) atmospheric simulation chamber to assess their practical viability. Density functional theory (DFT) calculations were employed to gain deeper insights into the sensing mechanism, aiding in understanding response times and identifying preferred sites for NO_2_ adsorption on the MoS_2_ nanosheet surface.

## Experimental Section

### Synthesis of Molybdenum Disulfide (MoS_2_)

A two-electrode configuration electrochemical cell was employed to exfoliate bulk MoS_2_ crystals. The cathode consisted of a MoS_2_ crystal, while a platinum foil served as the anode (see Supporting Information, Figure S1). The electrodes were immersed in an electrolyte solution containing 5 mg/mL of tetra-propyl ammonium (TPA) bromide dissolved in propylene carbonate. Upon applying a potential difference of 8 V for 30 min, TPA^+^ ions intercalate within the 2D crystal, causing its expansion.

### MoS_2_ Ink Formation

The expanded MoS_2_ crystals were treated by sonication for 5 min in a mixture composed of polyvinylpyrrolidone (PVP) at a concentration of 1 mg/mL, dissolved in dimethylformamide (DMF). After sonication, the solution was centrifuged for approximately 20 min and the sediment was discarded to separate unexfoliated crystals from the exfoliated nanosheets. The supernatant was centrifuged at 97 g for 1 h, and sediment was collected using our previously published protocol^17^ to obtain micron-sized MoS_2_ nanosheets. Following our previously published methodology, the nanosheets were washed with DMF and isopropanol (IPA) to remove residual PVP. The washed nanosheets were redispersed in IPA at a concentration of approximately 1 mg/mL (see Supporting Information, Figure S1), which was used for subsequent studies and investigations.

### Langmuir–Schaefer (LS) Method for Thin Film Deposition

A custom-built setup was used for the deposition of the nanomaterial, as published recently.^17^ A 250 mL beaker was filled with water until the water level completely covered the substrate on the holder. Approximately 2 mL of distilled *n*-hexane was added to the water in the beaker to create the liquid/liquid interface. The nanosheet ink was injected onto the liquid–liquid interface using a Pasteur pipet until a homogeneous film had formed and the substrate (∼2 cm^2^ PET) was lifted through the liquid/liquid interface to transfer the nanosheet layer onto the substrate. The wet substrate was allowed to dry at room temperature (∼4 h). Dry films were annealed at 120 °C for 1 h in an argon atmosphere to remove residual water from nanosheet junctions and interfaces.

### Sensor Device Fabrication

Gold (100 nm) was chosen to form contacts with MoS_2_ coatings and was deposited by a Temescal FC2000 through a shadow mask at a deposition rate of 0.15 nm/s. Sensor devices were fabricated using MoS_2_ ink by LS deposition on PET, giving a network thickness of 30 nm. Interdigitated electrodes (IDEs) with a gold thickness of 100 nm were evaporated onto the films to fabricate MoS_2_ sensor devices. The channel length of the sensor devices was fixed at 50 μm, with a channel width of 11 mm (see Supporting Information, Figure S1).

### Characterization of MoS_2_ Material

To understand the sensor mechanism, it is vital to know the size, morphology, thickness and layer numbers of the MoS_2_ being studied. Consequently, atomic force microscopy (AFM) was employed for data acquisition and analysis, using a Bruker Multimode 8 microscope, to investigate the size and thickness of MoS_2_. Measurements were performed in ScanAsyst mode in the air under ambient conditions using Al-coated Si cantilevers (OLTESPA-R3). The concentrated dispersion was diluted with IPA to optical densities <0.1 across the resonant spectral region. Drops of the dilute dispersions (20 μL) were deposited repeatedly on preheated (150 °C) Si/SiO_2_ wafers (0.25 cm^2^) with an oxide layer of 300 nm. After deposition, the wafers were rinsed with ∼15 mL of water and ∼15 mL of IPA and dried using compressed nitrogen. Typical image sizes were between 40 × 40 and 20 × 20 μm^2^ at scan rates of 0.4–0.8 Hz with 1024 lines per image. Previously published length corrections^18^ were used to correct lateral dimensions from cantilever broadening. The Raman spectroscopy measurements were conducted using the identical substrate used for AFM measurements. A WITec Raman spectrometer operating at 532 nm with a 50× objective lens was employed for this purpose, with an incident power of approximately 1 mW to minimize potential thermal damage during the analysis. X-ray photoelectron spectroscopy was recorded using a Kratos AXIS ULTRA spectrometer with an Al K_α_ X-ray gun.

### Characterization of MoS_2_ Sensor

The MoS_2_ coating substrates with gold-patterned films were characterized by scanning electron microscope (SEM) and X-ray photoelectron spectroscopy (XPS) to analyze surface morphology and chemical composition. Surface morphology was analyzed using a Quanta 650 SEM equipped with a Field Emission Gun. X-ray photoelectron spectra were obtained with MoS_2_ on PET using the Kratos AXIS ULTRA spectrometer with an Al K_∝_ source of 1486.58 eV.

Electrical measurements and gas sensing studies were performed using a gastight portable probe station (Nextron Instruments) interfaced with source measure units (SMUs, 2450 Keithley). The probe station has a volume of 100 cm^3^, is equipped with inlets and outlets for gas flows, and has a sapphire window which can be illuminated with UV radiation. The sensor configuration included a rotameter for regulating airflow and a digital mass flow controller (MFC) for controlling the flow of NO_2_ gas from the cylinder (1000 ppm of NO_2_ in air, BOC gases). For selectivity testing, four separate flow meters were used to handle NO_2_, ammonia (NH_3_), sulfur dioxide (SO_2_) and methane (CH_4_). Each flowmeter was connected to Swagelok valves, enabling precise control of the flow of each gas. The outputs from the flowmeters were connected to a gastight probe station for testing. Sensing tests were also conducted within an atmospheric simulation chamber made of FEP Teflon foil in the shape of a cuboid with a volume of 6.5 m^3^, described in detail elsewhere^19^ The FEP Teflon chamber was operated at atmospheric pressure using filtered compressed air (Zander KMA 75) and surrounded by 12 Philips TL05 (40 W) lamps (λ_max_ = 360 nm) and 12 Philips TL12 (40 W) lamps (λ_max_ = 310 nm). One side of the chamber was used to introduce gases, while the opposite wall accommodated the electrical sensors mounted via sample holders. Each sensor was bonded onto the 14-pin dual inline package (DIP) chip holder mounted on PTFE cylindrical tubes. Sensors were connected to a source meter via a 12-pin Fisher connector and a Bayonet Neill-Concelman (BNC) assembly. This setup ensured reliable data collection and electrical connections during the atmospheric simulation chamber testing.

The sensor response was calculated using eq 1(20):
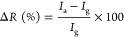
1

*I*_a_ and *I*_g_ represent the sensor current in air and the test gas (NO_2_), respectively. The response to the gases was evaluated at room temperature. Additionally, the responses were measured in humid air by passing synthetic air (20% oxygen balanced with nitrogen and <1 ppm of hydrocarbons) through a water bubbler.

### Computational Methodology

Data were obtained using the Density Functional Theory (DFT) code Vienna Ab Initio Simulation Package (VASP),^21^ employing a plane-wave basis (with an energy cutoff of at least 400 eV), projector augmented waves (PAWs)^22^ and the generalized gradient approximation (GGA) Perdew–Burke–Ernzerhof^23^ exchange–correlation (xc) functional. Additionally, nonbonding van der Waals interactions were considered utilizing the DFT-D3 scheme.^24^ To simulate the impact of molecular adsorption on 2D materials, monolayers were employed in supercell geometries (with 90 atoms for MoS_2_ in the pristine, i.e., adsorbate-free, state). Structural representations were generated using the software package VESTA.^25^

## Results and Discussion

### Structural and Compositional Analysis of MoS_2_

**Figure 1:**
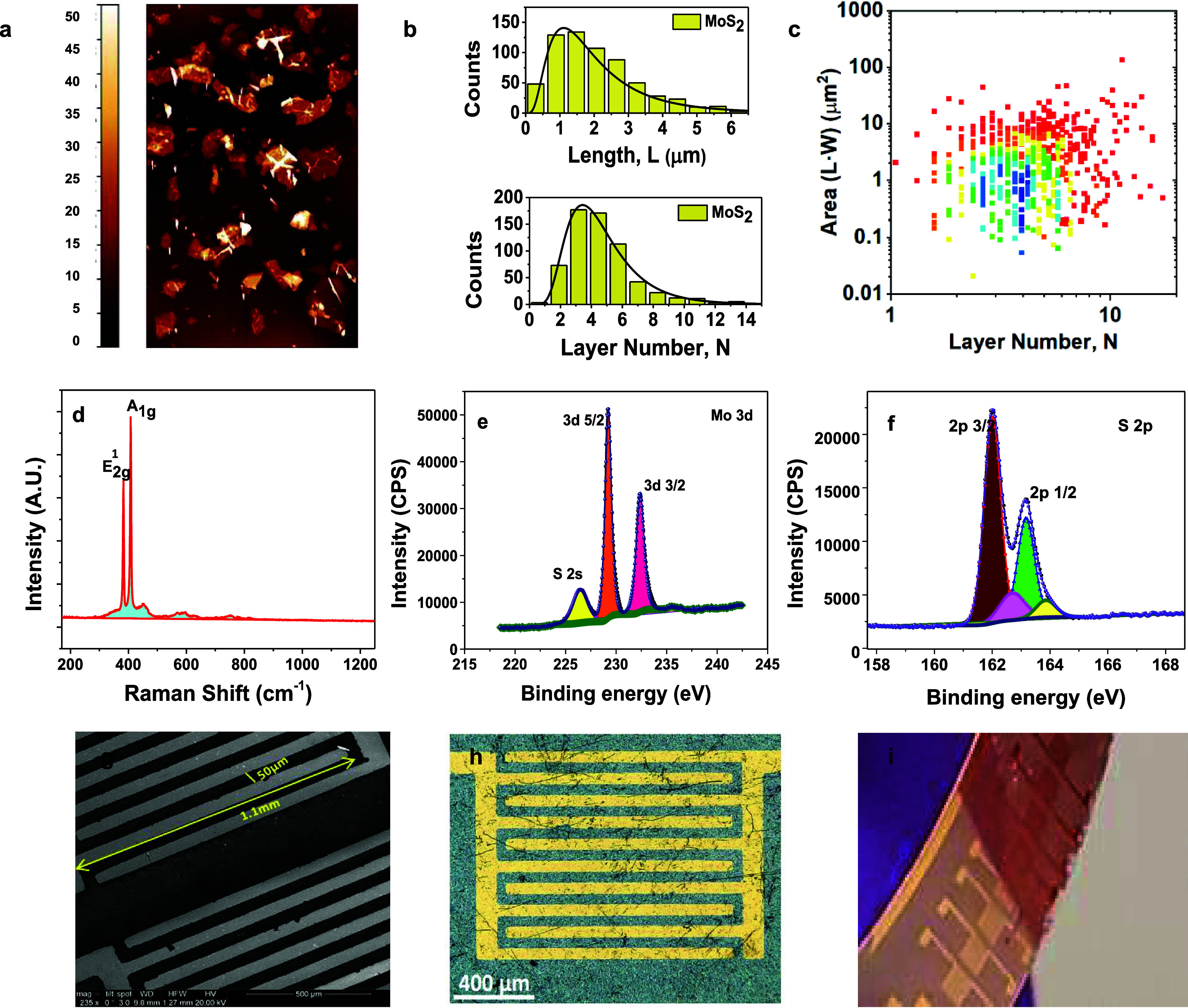
(a) Atomic force micrograph of MoS_2_ flakes, (b) statistical analysis of flake length and layer numbers, (c) statistics distribution of layer number with the area of the film, (d) Raman spectrum of the exfoliated MoS_2_. X-ray photoelectron spectra of (e) Mo 3d core level spectra and (f) S 2p core level spectra. SEM image, optical microscope image, and the flexibility of the fabricated sensor device on the PET substrate are shown in (g), (h), and (i), respectively.

Statistical AFM analysis was undertaken on the MoS_2_ nanosheets to ascertain their dimensions. Figure 1a shows the topography of a drop-cast sample of MoS_2_ nanosheets. Most flakes ranged between 1 and 2 μm, with a mean layer count of 4, as illustrated in the length vs layer count distribution plot (Figure 1b). Figure 1c illustrates the statistical distribution of the number of layers vs nanosheet area, indicating that the nanomaterial predominantly consists of MoS_2_ nanosheets with layer numbers ranging from 1 to 11 layers and an area between ∼0.05–100 μm^2^. The MoS_2_ films comprised a continuous network of MoS_2_ flakes with a lateral dimension of approximately 1–2 μm, as shown in Figure S2 (see Supporting Information). The relationship between the dimensions of the gas sensing material and the sensor response is governed by a parameter known as the Debye length. When the Debye length is comparable to the material thickness, the entire material is influenced by gas adsorption, enhancing its sensing capability. In this study, the MoS_2_ flakes are 1–11 layers thick, with their thickness consistently below the Debye length for all morphologies (a few nanometers), which is ideal for gas applications.

Figure 1d illustrates the Raman spectrum obtained from the electrochemically exfoliated 2D MoS_2_ flakes. A typical Raman spectrum showed the E^1^_2g_ mode at 384 cm^–1^, corresponding to the in-plane vibration of Mo–S atoms.^26^ Additionally, the appearance of the A_1g_ Raman mode at 409 cm^–1^ corresponds to the out-of-plane vibration of Mo–S atoms.^26,27^ The wavenumber difference between the E_2g_ and A_1g_ modes is typically 19.5 cm^–1^ for a monolayer. This difference increases with an increase in the layer number due to interlayer coupling. Hence a difference of 25 cm^–1^ corresponds to MoS_2_ with more than four layers, as suggested by AFM data of layer number vs area density in Figure 1c.^28^

To understand the chemical composition, surface states and oxidation states of Mo and S in the MoS_2_ flakes, X-ray photoelectron spectroscopy (XPS) was performed on MoS_2_ films deposited using the LS method onto PET substrates, as presented in Figure 1e,f and Table S1 (see Supporting Information). Figure 1e shows the deconvoluted Mo 3d core level spectra, revealing a doublet consisting of 3d5/2 and 3d3/2 peaks at 229.2 and 232.4 eV, respectively. An additional peak at a lower binding energy can be attributed to the S 2s peak. The S 2p core level spectra depicted in Figure 1f show four peaks deconvoluted into two doublets. The first two peaks at 162.0 and 163.2 eV represent the spin–orbit doublets corresponding to 2p3/2 and 2p1/2 of sulfide (S^2–^). Meanwhile, the other doublets at 162.7 and 163.9 eV can be assigned to 2p3/2 and 2p1/2 of S_2_.^2−29^ The binding energy positions of Mo and S atoms are indicative of MoS_2_.^30^ However, the atomic percentage ratio between Mo^4+^ and S^2–^/S_2_^2–^ is 1:1.8 (±0.1). This deviates from the stoichiometric ratio (1:2) for ideal MoS_2_, indicating the fabrication of the sulfur-deficient MoS_2_ nanosheets prepared by the electrochemical exfoliation method, as confirmed by EDX analysis (Figure S2). Detailed XPS quantification is given in Table S1 (see Supporting Information). The overall device configuration, transparency, morphology and flexibility of the MoS_2_ sensor device are shown in Figure 1g,h, respectively (additional images are shown in Figures S1 and S2 in the Supporting Information). Figure 1g presents an SEM image of a PET substrate uniformly coated with MoS_2_, featuring interdigitated Au electrodes with a finger width of 50 μm and a length of 1.1 mm. The figure illustrates the uniform distribution of MoS_2_ across the substrate, highlighting the effectiveness of the ink jet printing technique in achieving uniform thin films. Figure 1h highlights the flexibility of the fabricated transparent sensor device. For detailed information on the synthetic method, the structure of MoS_2_ 2D flakes and the fabrication of similar device structures, we refer readers to our previous publication.^17^

### Electrical and Gas Sensing Measurements

**Figure 2:**
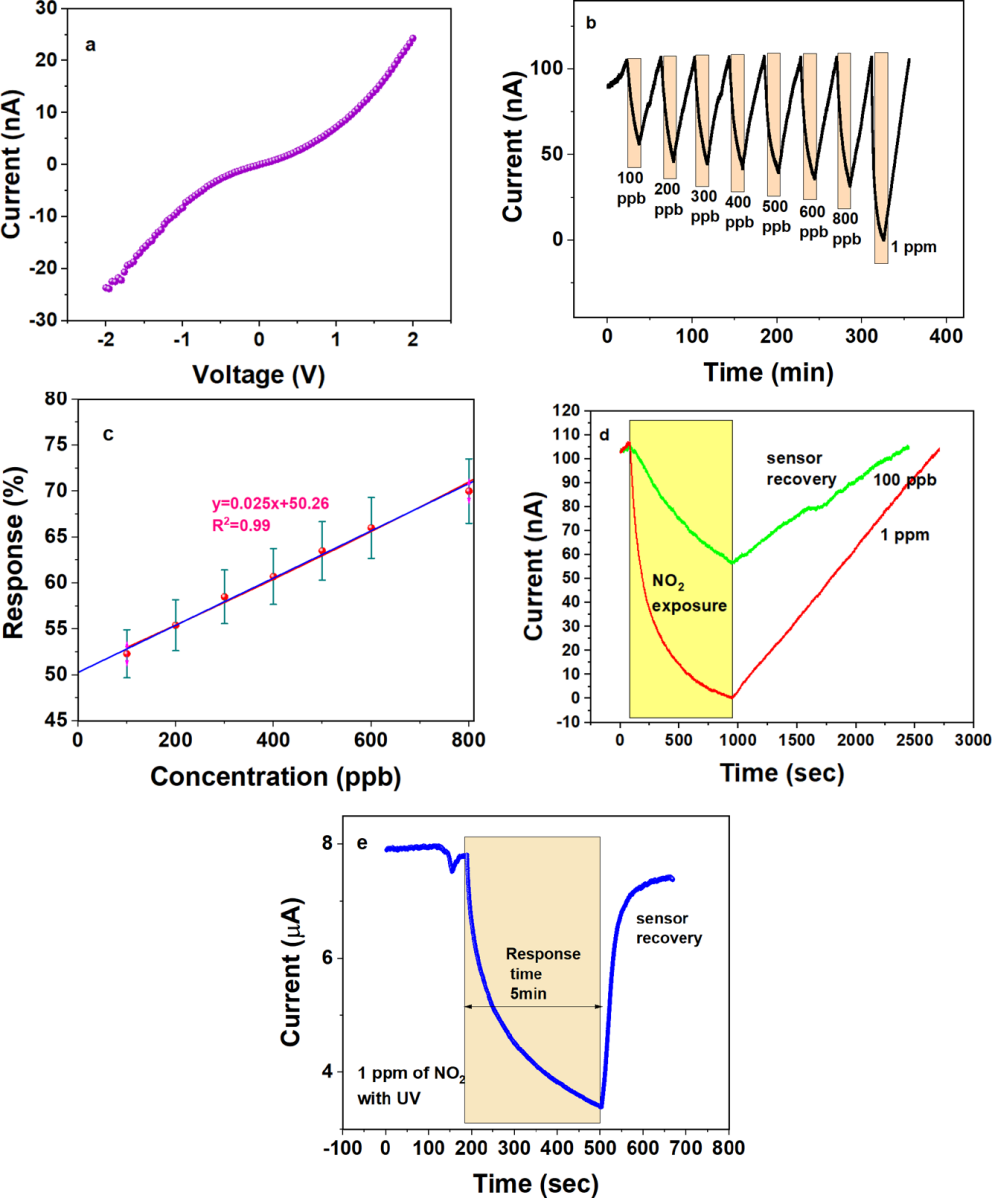
(a) Two-terminal device’s current–voltage (*I*–*V*) curve at room temperature. (b) Transient response of the device for various concentrations of NO_2_ gas at room temperature. (c) Response curve from a lower to high concentration of NO_2_ gas. (d) Response curve for evaluating the response and recovery times and (e) response curve for 1 ppm of NO_2_ under UV light in a probe station.

Initial electrical measurements and gas sensing experiments were conducted on the fabricated sensor devices (see Experimental Section for device detail). Figure 2a illustrates the current–voltage (*I*–*V*) characteristics of an MoS_2_ device. These measurements were performed using a voltage sweep of ±2 V in a two-terminal configuration setup at room temperature. The device exhibits nonlinear *I*–*V* characteristics. The sensor response to NO_2_ at room temperature was assessed using the same device by biasing the device at 1 V. Operating in the nonlinear region amplifies sensitivity, as resistance changes cause more pronounced current fluctuations for a given voltage shift. This region generally exhibits an enhanced response to gas adsorption compared to linear regions, enabling the detection of lower gas concentrations.^31^ By carefully selecting an operating point within the nonlinear region, it may be feasible to identify specific gases based on their unique interactions with the sensor material. However, operating in this region can occasionally introduce stability and variability challenges due to device inconsistencies, temperature fluctuations, or noise.

Two-terminal electrical measurements were carried out on the MoS_2_ devices in various environments: ambient air, synthetic air and a mixture of NO_2_ with synthetic air. Initially, the device was exposed to 10 L of synthetic air for 30 min to establish a baseline current. Subsequently, the device was exposed to NO_2_ gas diluted with air to achieve a concentration of 100 ppb for 10 min. Following this, the NO_2_ supply was turned off, allowing the device to return to its initial state. This process was repeated regularly, with different concentrations of NO_2_ gas introduced into the gastight probe station every 20 min. Each concentration was allowed to react with the sample for 10 min, ranging from 100 ppb to 1 ppm.

The transient sensor response to NO_2_ gas at room temperature was analyzed, as shown in Figure 2b. The increase in resistance or decline in current upon exposure to NO_2_ suggests electron entrapment by the oxidizing gas NO_2_. The sensor response was quantified using eq 1 and is depicted in Figure 2c, with an error bar of 5% of the sensor response. The figure shows that the response demonstrates a linear relationship with increasing NO_2_ gas concentration. Notably, a relative response of approximately 50 and 90% was observed for concentrations of 100 ppb and 1 ppm of NO_2_, respectively.

MoS_2_ exhibits a strong adsorption coefficient for NO_2_ gas molecules, effectively absorbing enough NO_2_ even at room temperature. This property facilitates and promotes the room temperature sensing of NO_2_ gas using MoS_2_. The observed decrease in current upon exposure to NO_2_ can be attributed to the n-type semiconductor behavior of the material, which is influenced by the presence of inherent anionic sulfur vacancies. These vacancies are known to exist due to their relatively low formation energy and are typically observed in MoS_2_, as reported in the literature.^32^ Positioned above the valence band maxima, these vacancy sites act as shallow donors, readily donating electrons to the conduction band of MoS_2_ and consequently contributing to its n-type behavior. The MoS_2_ network arrangement, characterized by interconnecting flakes, exposes reactive edge sites that are significantly more chemically active than the basal planes within the material’s structure.^33^ As a result, these exposed edge sites tend to adsorb ambient oxygen and moisture in conjunction with the vacancy sites. This adsorption process leads to the incorporation of oxygen molecules as O^2–^ ions at room temperature,^34^ where an electron is extracted from the surface by eq 2.
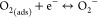
2

The adsorbed oxygen diminishes the surface’s availability for interaction with the NO_2_ target gas. Nonetheless, when exposed to the oxidizing NO_2_ gas, the sensor experiences a decrease in current and an increase in resistance. This occurs due to the formation of an electron depletion layer on the MoS_2_ surface,^30^ as described by eq 3. Subsequently, charge transfer occurs from the surface to the NO_2_ analyte, reducing current.
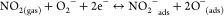
3

The sensor response with varying NO_2_ concentrations was analyzed through linear fitting, as shown in Figure 2c, with a high coefficient of determination (*R*^2^ = 0.99) to define the limit of detection (LOD). The LOD signifies the concentration at which a sensor’s response becomes distinguishable from the noise level and can be determined using eqs 4 and 5. To evaluate the noise level, the root-mean-square noise (RMS_noise_) was calculated by averaging the signal over a span of 10 data points before the onset of NO_2_ exposure and is given by eq 4(35):
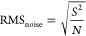
4

*N* is the number of data points, and *S* is the sensor response. The calculated RMS_noise_, i.e., the standard deviation of the sensor response, was found to be 1.79 × 10^–8^. The LOD of the MoS_2_ sensors was calculated using eq 5.
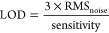
5

From the sensitivity calculated from Figure 2c, the theoretical LOD of the sensor, as calculated based on eq 5, was estimated as 1.9 parts per quadrillion (ppq) or 1.9 × 10^–15^. Our investigation suggests that the sensor response and theoretical LOD of our printed flexible MoS_2_ sensor are superior compared to bare, i.e., without doping or hybridization, MoS_2_ sensors reported in the literature, as presented in Table 1. Notably, the sensor response of 90% for 1 ppm toward electrochemically exfoliated MoS_2_ at room temperature surpasses previously reported response from MoS_2_ nanowire networks, i.e., ∼6% observed at ∼1 ppm of NO_2_.^36^ Many previously reported MoS_2_-based NO_2_ sensors are fabricated using the expensive EBL method and on rigid substrates, unlike the methods and substrates used in our NO_2_ sensor.

**Table 1:** Comparative Analysis of MoS_2_-Based NO_2_ Sensors with the Current Study

material and substrate	*T* (°C)	conc (ppm)	response (%)	response time	recovery time	LoD	ref
4 nm MoS_2_on PET	RT	1.2	6.1	30 min	30 min	1.2 ppm	(39)
MoS_2_ layer on SiO_2_	RT	100	54	180 s	600 s	100 ppm	(40)
MoS_2_ hollow sphere on SiO_2_	150	500	88.3	80 s	225 s	0.5 ppm	(41)
MoS_2_ nanoflower on SiO_2_	150	5	30	125 s	485 s	1.2 ppm	(12)
MoS_2_ nanowire network on SiO_2_	60	5	18	16 s	172 s	1 ppm	(36)
MoS_2_ on SiO_2_	RT	100	27	4.5 min	n/a	5 ppm	(34)
n-type MoS_2_ on SiO_2_	200	1		41 min	39 min	20 ppb	(30)
MoS_2_ nanoflakes grown on In_2_O_3_ microtubes as powder	RT	0.15	∼2	7 s	182 s	150 ppb	(42)
Single flake MoS_2_ by EBL on SiO_2_	RT	0.001	5.1	50 ms	75 ms	1 ppb	(35)
MoS_2_ on SiO_2_	RT(UV)	100	35	29 s	350 s	5 ppm	(34)
Langmuir–Schaefer MoS_2_ network on PET	RT	1	98	10 min	20 min	100 ppb	This work
Langmuir–Schaefer MoS_2_ network on PET	RT(UV)	1	96	300 s	60 s	100 ppb	This work

The sensor response to high NO_2_ concentrations, ranging from 5 to 50 ppm, is shown in Figure S4 (see Supporting Information). A response above 90% for 1 ppm of NO_2_ at room temperature, highlights device effectiveness across a broad concentration range spanning from 100 ppb to 1 ppm. Furthermore, the sensor response and recovery times, i.e., the duration required for a sensor to reach a maximum point and decay to the initial point,^24^ are computed using the response curve and presented in Figure 2d. At a lower concentration of 100 ppb, the sensor did not reach a steady, saturated state even after 10 min of NO_2_ exposure (Figure 2d). In contrast, at 1 ppm of NO_2,_ the sensor nearly reached a steady state within the same time frame (Figure 2d). This behavior indicates that the surface adsorption of the gas molecules increases linearly with rising gas concentration. The complete recovery time for both cases was estimated to be 20 min (Figure 2d), which is relatively slow due to the higher binding energy of NO_2_ on the MoS_2_ surface, as supported by DFT calculations for this specific concentration of Mo and S. The sensor’s inability to reach saturation after 10 min at 100 ppb suggests that either a higher concentration of gas molecules or a longer exposure time is required for surface saturation. The observed desorption after 10 min is attributed to the absence of NO_2_ in the probe station once the gas flow is turned off. Temperature, humidity, and gas concentration influence the sensor’s adsorption and desorption processes. In transient responses, nonsteady-state conditions can arise due to imbalances between adsorption and desorption, driven by changes in gas concentration. This behavior, including desorption occurring before a steady state is reached, has been previously reported in the literature.^37,38^

To speed up the recovery time, NO_2_ sensing experiments were conducted under UV illumination at a bias voltage of 1 V, both in the probe station and the chamber. UV-activated gas sensors utilize UV light to generate electron–hole pairs, accelerating gas molecule desorption. A mercury pen ray lamp emitting UV radiation at 254 nm in the probe station was directed through the sapphire window onto the sensor. Following a stabilization period of 20 min under a flow of 5 SLPM of air, the response of the illuminated sensor to 1 ppm of NO_2_ introduced for 5 min was measured, as depicted in Figure 2e. The UV light remained active throughout the experiment. The response and recovery times were estimated to be approximately 5 min and less than 60 s, respectively, in UV light. The figure shows that UV light improves response and recovery times compared to without it. Furthermore, as illustrated in Figure 2e, the sensor fully recovers to its initial state when exposed to UV light. When NO_2_ adsorbs onto the MoS_2_ surface, it extracts electrons, reducing the concentration of significant carriers in n-type MoS_2_. Under UV light, photons generate electron–hole pairs, providing an additional source of electrons and enabling a constant current flow in the sensor. The photogenerated carriers increase charge density and accelerate surface reactions by enhancing the availability of active sites and aiding the desorption of preabsorbed oxygen ions. This increases the adsorption and desorption rates, thereby improving the sensor’s response. Photogenerated holes also react with adsorbed NO^–^, facilitating the desorption of NO_2_ and significantly improving the recovery rate. The recovery and response times for the MoS_2_ sensor are notably faster than previously reported MoS_2_ sensors for NO_2_ (see Table S2 in the Supporting Information). Our printed MoS_2_ sensor demonstrates competitive sensing performance across key parameters, including response time, recovery time, and detection limit detection range, under the influence of UV light. This performance is particularly notable given the simplicity of the fabrication process, a nonlithographic printing approach, and the use of straightforward materials without additional doping, heterostructures, or functionalization.

To understand the processes and evaluate the efficiency of NO_2_ sensing using electrochemically exfoliated MoS_2_, we conducted DFT calculations to investigate interactions between NO_2_-related species and a MoS_2_ monolayer. We considered various concentrations of sulfur vacancies (S_V_), ranging from pristine MoS_2_ up to 10% of sulfur vacancies, following the nonstoichiometric configuration observed for the electrochemically exfoliated MoS_2_ samples used in this study (see Supporting Information, XPS data in Table S1). Furthermore, we explored the interaction of NO_2_-related species with the site where a sulfur vacancy is replaced by oxygen (referred to as a substitutional O site). Our findings suggest a strong energetic preference (of about 5.00 eV) for an oxygen molecule to dissociate on an isolated sulfur vacancy, creating a substitutional oxygen site within MoS_2_ and an oxygen adatom, i.e., O that sticks on the surface (in this case, on a S atom of MoS_2_) can be called an adatom, bonded to a sulfur atom. Likewise, a significant energy gain (8.18 eV) occurs when an oxygen molecule passivates two sulfur vacancies, creating two oxygen substitutional sites. These high reaction energies suggest that oxygen atoms will occupy most sulfur vacancies in the nonstoichiometric MoS_2_ samples.

**Figure 3:**
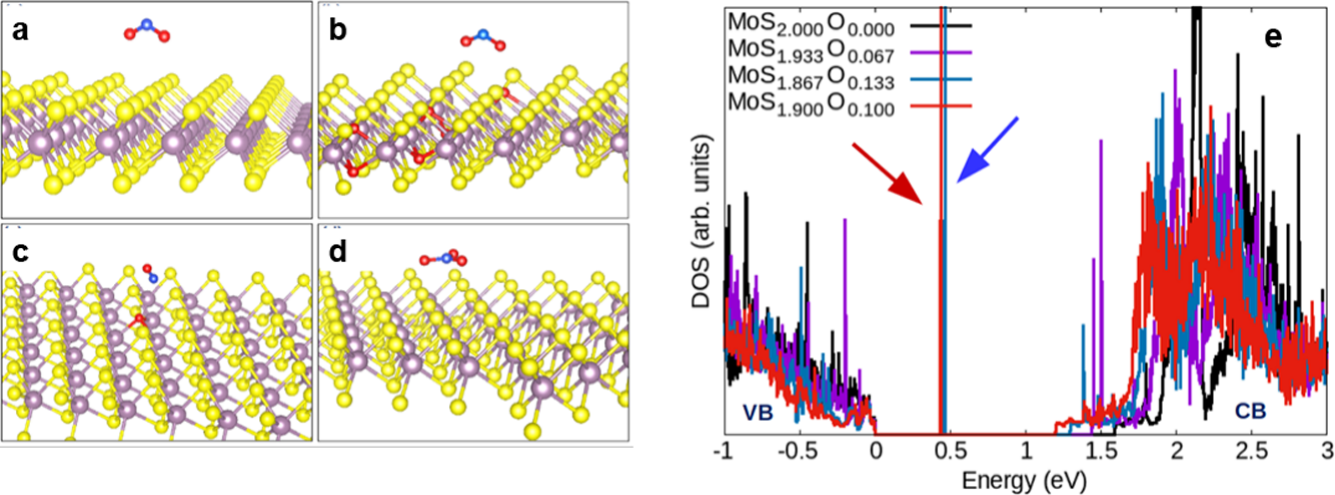
(a, b) Physisorption of a NO_2_ molecule on (a) a MoS_2_ layer and (b) a layer with Mo:S:O ratio as 1:1.8:0.2 layer. (c) Passivation of a sulfur vacancy by an oxygen atom and a physisorbed NO molecule on a MoS_2_ layer. (d) Nitrate radical (NO_3_^·^) physisorbed on a MoS_2_ layer (Mo: purple, S: yellow, O: red, N: blue spheres) and (e) electronic DOS for configurations of a NO_2_ molecule on a MoS_2_ layer (black line), a layer with Mo:S:O ratio as 1:1.93:0.06 (purple line), a layer (blue-black line) and a layer Mo:S:O ratio as 1:1.8:0.2 (blue line). Energy zero point is set at the highest occupied state. VB (CB) is the layer’s valence (conduction) band. Arrows indicate the electronic trap states within the energy band gap in the nonstoichiometric case.

As an electron acceptor, NO_2_ molecules tend to capture electrons from neighboring systems. In the current context, this capture mechanism can be facilitated by introducing NO_2_-related empty states within the band gap of MoS_2_. Initially, our findings indicate that a NO_2_ molecule physisorbed on a pristine MoS_2_ layer (as shown in Figure 3a) remains stable (with a stability of 0.60 eV) against dissociation into a physisorbed NO molecule and an oxygen adatom. When such a physisorbed NO_2_ molecule is situated on a fully stoichiometric MoS_2_ layer, the electronic density of states (DOS) plot (depicted as a black line in Figure 3e) reveals no states within the band gap of MoS_2_. However, a shift occurs when the MoS_2_ layer is nonstoichiometric, with oxygen atoms passivating its sulfur vacancy (S_V_). Specifically, for layers with the elemental ratios of Mo:S:O as 1:1.9:0.13 and 1:1.8:0.2 respectively, the physisorbed NO_2_ molecule (depicted in Figure 3b) generates empty states within the band gap of the material, as shown in the DOS plots of Figure 3e with blue and red lines. In any n-type sample, these vacant gap states can trap electrons from the material’s conduction band, thereby decreasing the current, as observed, and facilitating the detection of NO_2_.

Conversely, our investigation reveals that the interaction of a NO_2_ molecule with an S_V_ can also result in the passivation of the defect by an O atom, leading to the formation of an NO molecule (see Figure 3c). This reaction is strongly exothermic, with an energy decrease of 2.12 eV. Notably, this resultant product configuration serves as an electronic acceptor, featuring an empty state below the conduction band of MoS_2_. Although this state functions as a shallow trap capable of exchanging electrons with the conduction band, its contribution to NO_2_ sensing is not as pronounced as the deep trap state observed in the NO_2_ physisorption discussed above. Furthermore, it is worth noting that the NO molecule could also react with O adatoms, potentially formed on sulfur nonstoichiometric samples via mechanisms such as the oxygen dissociation discussed above. The result of this latter reaction is the reformation of a NO_2_ physisorbed molecule with an energy gain of 0.60 eV.

Based on the results of the DFT calculations, we have also identified a third potential mechanism which enables the sensing of NO_2_ through the capture of electrons. Specifically, we found that NO_2_ molecules can react with oxygen atoms to create nitrate species. Under neutral conditions, this process is energetically unfavorable, incurring an energy penalty of 0.40 eV. However, in the presence of an extra electron, characteristic of n-type samples, the reaction becomes exothermic with an energy gain of 0.26 eV. As a result, a nitrate radical anion (NO_3_^·–^) is formed (illustrated in Figure 3d), which means that a negative charge is formed, indicating that an electron originating from the material’s conduction band of MoS_2_ is once again captured, thus decreasing the current. To summarize the DFT main findings, we have identified three primary mechanisms for the detection of NO_2_ through electron trapping of electrons: (i) the physisorption of NO_2_ molecules on oxygen-passivated defective MoS_2__–_*_x_* layers, (ii) the passivation of sulfur vacancies of MoS_2–*x*_ through the dissociation of molecules and the formation of physisorbed nitrogen monoxide and (iii) the formation of nitrate radical anions.

The predominant sensing mechanism is the physisorption of NO_2_ molecules on oxygen-passivated, defective MoS_2–*x*_ layers. DFT calculations reveal that in sulfur-deficient MoS_2_ layers, physisorbed NO_2_ molecules introduce empty states within the material’s band gap, as shown in the DOS plots in Figure 3e (blue and red lines). These empty states are absent in stoichiometric MoS_2_. XPS and EDX measurements (Figures 1e,f and S2 in Supporting Information) confirm sulfur deficiency in the electrochemically exfoliated MoS_2_, aligning with the DFT results. These findings highlight the importance of sulfur-deficient MoS_2_ in enabling efficient charge transfer and sensing upon interaction with NO_2_. Since the active product species are primarily physisorbed, some desorption is expected when the supply of NO_2_ is interrupted, corresponding with the recovery phase observed in the experiments. The sensor’s operating temperature is primarily determined by the availability of active sites on the surface and the target gas. For example, MoS_2_ sensors designed for H_2_ detection typically require elevated temperatures and do not operate at room temperature. However, MoS_2_ exhibits a high adsorption coefficient for NO_2_, enabling sufficient adsorption even at room temperature, which allows room temperature NO_2_ sensing. Additionally, MoS_2_ surfaces with active sites, such as defects, dangling bonds and other imperfections, provide effective adsorption sites that facilitate charge carrier exchange. These active sites in defective MoS_2_ make it more suitable for room temperature sensing than defect-free MoS_2_. In addition, specific mechanisms discussed above may be influenced by UV light or the presence of other species, such as water molecules. For example, UV light can facilitate the passivation of sulfur vacancies by breaking up oxygen molecules into atomic constituents, thus generating oxygen that aids the formation of nitrate radicals. In addition, UV light can play a role in dissociating H_2_O molecules into H^·^ and HO^·^ radicals, which can subsequently react with physisorbed nitrate and nitrogen dioxide species, respectively, to accelerate the recovery phase by forming inactive HNO_3_ molecules.

**Figure 4:**
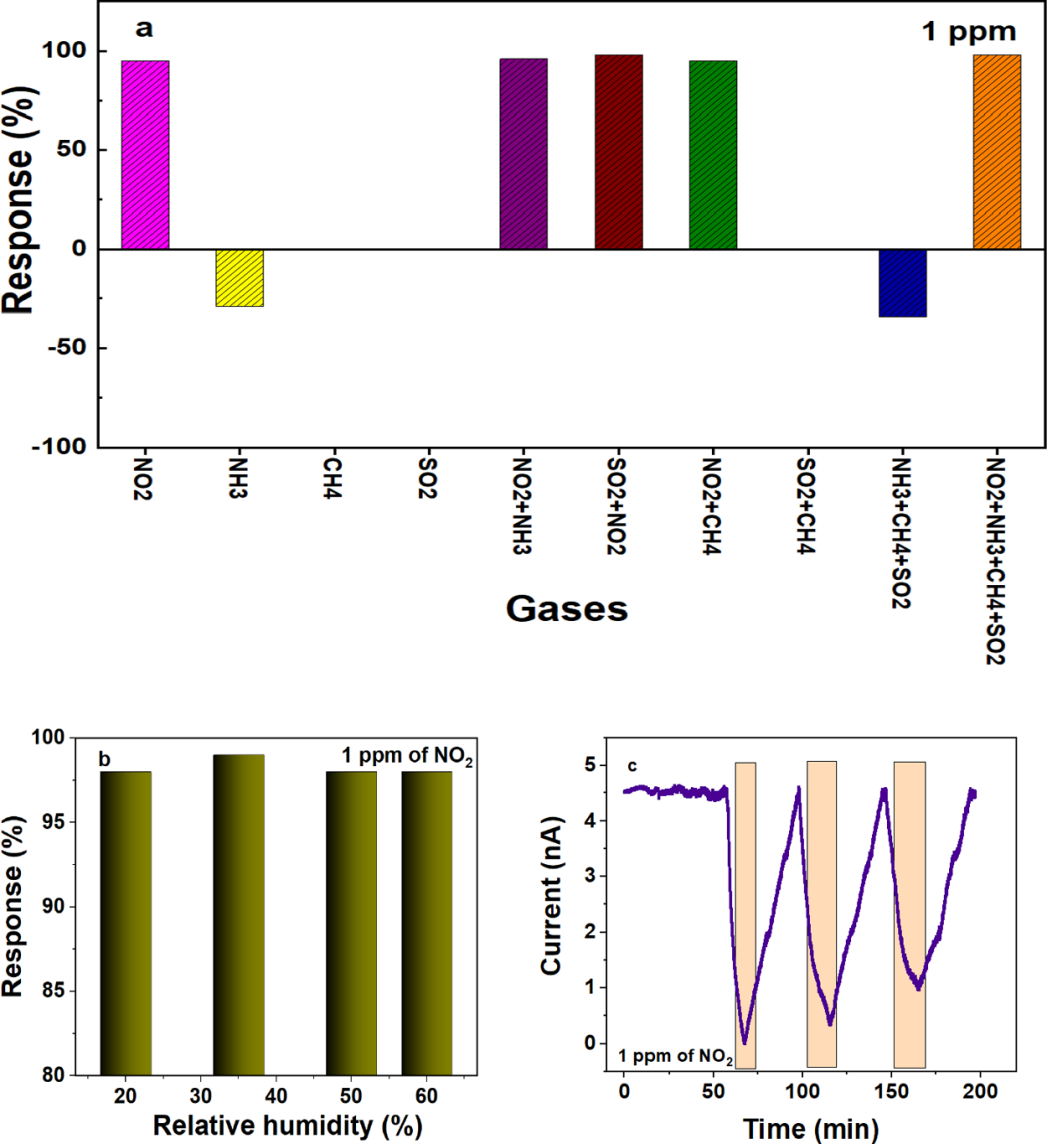
(a) Bar graph illustrating response values calculated for all combinations of gas mixtures. (b) Response values for 1 ppm of NO_2_ at various relative humidity (RH) levels at room temperature. (c) Response curve of the MoS_2_ sensor for three pulses of 1 ppm of NO_2_.

Furthermore, the sensor’s selectivity to NO_2_ at room temperature was investigated by assessing its response to other prevalent air pollutants such as NH_3_, SO_2_, and CH_4_. The cross-sensitivity and selectivity toward NO_2_ were examined by measuring the sensor response in 1 ppm of NO_2_, NH_3_, SO_2_, and CH_4_ individually and combining these gases (see Figure 4a). Detailed response curves for each gas and different gas combinations are provided in Figure S5 (see Supporting Information). The sensor showed no response or a distinct response, e.g., a positive response, indicating an increase in current toward NH_3_ gas to all other pollutants. Furthermore, to assess the influence of secondary products from these gas mixtures, the sensor response was evaluated in the presence of mixtures containing each pollutant at a concentration of 1 ppm. As shown in Figure S5 (see Supporting Information), the response value approaches ∼100% when NO_2_ is present in any gas combination, indicating its predominant contribution toward the sensor response. Although the sensor showed a positive response in the presence of 1 ppm of NH_3_, when mixed with 1 ppm of NO_2_, the overall response resembled that of NO_2_ alone. This suggests that NH_3_, despite being a reducing analyte, does not interfere with NO_2_ sensing. Figure 4a depicts the sensor response in a mixture containing all four gases simultaneously. The response of the MoS_2_ sensor toward NO_2_ remains consistent with that observed during individual exposure to 1 ppm of NO_2_. This indicates that any potential byproducts generated in the gas mixtures do not interfere with the sensor signal.

Understanding humidity’s impact is crucial for practically applying gas sensors. Typically, sensor devices exhibit degradation, manifesting as a decrease in the sensor signal, after prolonged exposure to water vapor. Therefore, the effect of relative humidity (RH) calculated from the concentration and the bubbler temperature on the device was investigated over 20–75%. Humidified air was generated by passing five standard liters per minute (5 SLPM) of synthetic air through a water-filled bubbler. This bubbler was maintained at a specific temperature (50–80 °C) to achieve the desired RH. The response of the MoS_2_ devices was evaluated in the presence of 1 ppm of NO_2_ under various humidity conditions. Figure 4b shows that the device response remained unaffected across different RH levels. This observation suggests the absence of significant reactions between the sensor surface and the water molecules, thereby preserving sensor performance under diverse humidity conditions.

The stability of the device was assessed by measuring its response at the highest and lowest concentrations of NO_2_. A continuous series of three pulses, each at a concentration as low as 100 ppb, was passed over the device, and the responses are shown in Figure S6a (see Supporting Information). Figure 4c also shows the response curve for three consecutive pulses of 1 ppm of NO_2_. In both cases, the responses fall within the error range of ±5% of the total response. To address the potential degradation of the devices over time due to constant exposure to NO_2_ gas, their stability after 2 months of initial NO_2_ exposure across a range of concentrations from 100 ppb to 1 ppm was studied (see Supporting Information, Figure S6b). The response at 100 ppb remained at 40%, compared to an initial response of approximately 50%, while the response at 1 ppm remained consistent at 96%, mirroring the initial response. This implies a reduction in response of less than ±10% over two months. Consequently, it can be inferred that the device remains stable for continuous use for at least two months.

**Figure 5:**
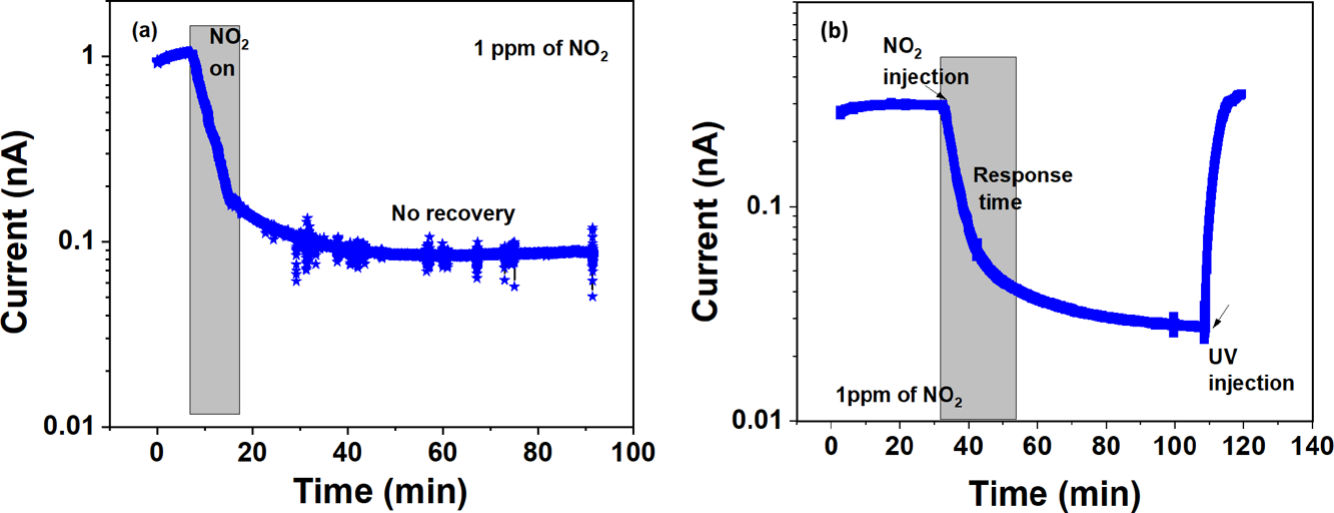
Transient response of MoS_2_ to 1 ppm of NO_2_ in the atmospheric simulation chamber. Under dark conditions (in part (a)), no sensor recovery was observed, whereas a fast recovery was achieved with UV light (part (b)).

To replicate sensor performance under more realistic atmospheric conditions, testing was conducted in a large volume atmospheric simulation chamber (see Supporting Information, Figure S7). To evaluate the mixing ratio of NO_2_ within the chamber, a fixed concentration of 1 ppm of NO_2_ was introduced into the chamber with varying injection times of 1, 2, 4, and 10 min, and the resulting gas concentrations were measured via NO_2_ monitor. Figure S9 gives the mixing ratio for the chamber used. It is noted that a minimum mixing time of 1 h was needed to achieve the desired concentration of NO_2_ within the chamber. Given that the performance of the MoS_2_ sensor was evaluated at an optimal concentration of 1 ppm of NO_2_ in the probe station, the sensor response for NO_2_ within the atmospheric chamber was observed for the same concentration. Following the installation of the sensor in the chamber, stabilization of the electrical signal was achieved while flushing the chamber with filtered compressed air at a rate of 50 LPM for 60 min. The flushing was stopped, and sufficient NO_2_ was added to the chamber at a rate of 20 LPM to achieve a mixing ratio of 1 ppm for 10 min. Characterization and calibration of the atmospheric chamber are detailed in the Supporting Information. The electrical response behavior of the sensor during the addition of NO_2_ was similar to that observed in the probe station. Figure 5a shows that the current dropped rapidly as NO_2_ was added. After an initial period of gas mixing in the chamber and the sensor adjusting to the presence of the gas, the current remained steady for the remainder of the experimental test. The sensor did not start to recover even though the chamber was continually flushed with compressed air at a rate of 20 LPM throughout the experiment. Data from atmospheric chamber simulations was collected to explain the sensor’s nonrecovery and confirm the presence of residual NO_2_ concentrations in the chamber after NO_2_ purging. The variation in concentration of gases within the chamber over time, for a fixed purge concentration, was calculated by considering various factors, including the emission rate of the Teflon wall of the chamber, dilution rate constant, chamber volume, purge air concentration and removal constant. The conditions governing this concentration variation are provided in eq 6. Several trials were conducted to assess the wall loss and observe the decay of NO_2_ concentration after stopping the NO_2_ purge. Even after stopping the purge, an increase in NO_2_ concentration was observed, indicating that the chamber walls act as a source of NO_2_ (Figure S8a). The decay curve shown in Figure S8b further verifies the presence of NO_2_ in the chamber after the injection was stopped. NO_2_ concentration in the chamber remains approximately 90 ppb after 3.7 h of halting the NO_2_ purge of 100 ppb.
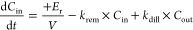
6

*E*_r_, *k*_rem_, *k*_dill_, *C*_in_, and *C*_out_ are the emission rate, the removal process constant, the dilution rate constant and the chamber and purge air concentration, respectively. Even at a low concentration of 100 ppb of NO_2_, the complete desorption of NO_2_ from the chamber took significant time. After 3 h, the NO_2_ concentration remained around 94 ppb (Figure S8b), preventing the MoS_2_ sensor from achieving recovery in the atmospheric chamber (Figure 5a). The response time, determined through exponential fitting, was calculated to be 20 min.

To accelerate the sensor’s recovery in the atmospheric simulation chamber, experiments were conducted with UV radiation centered around 360 nm during the recovery period. The sensor’s response to 1 ppm of NO_2_ is presented in Figure 5b. The calculated sensor response in the chamber was 90%, closely aligned with the response observed in the probe station setup. A significant improvement in the recovery time to 7 min was observed in the atmospheric chamber with UV irradiation.

## Conclusions

In summary, we propose an innovative technique involving electrochemically exfoliated MoS_2_, forming 2D networks, which were further explored for their ability to detect NO_2_ at parts per billion (ppb) levels at room temperature. When exposing the sensors to UV light, significant enhancements in response time and recovery times were observed. The printed MoS_2_ sensor demonstrates competitive sensing performance across multiple parameters, such as response time, recovery time, limit of detection and detection range, under UV illumination. This performance is impressive given the ease of fabrication (a nonlithographic, printed approach) and simplified material design, requiring no additional doping, heterostructures or functionalization. Notably, within a sizable atmospheric simulation chamber, the sensors exhibited a remarkable 90% response to 1 ppm of NO_2_. DFT calculations identified three main mechanisms for NO_2_ detection, involving the trapping of electrons due to physisorbed NO_2_ and NO molecules on the MoS_2_ surface, with pertinent calculated binding energies aligning well with experimental findings. The MoS_2_ sensors displayed significant selectivity toward NO_2_ in the presence of other common air pollutants and water vapor. No dependence on RH in the range 20–75% was observed. Importantly, this study marks the first reported instance of electrical NO_2_ sensing measurements in a large atmospheric simulation chamber, adding relevance, uniqueness, and significance to real-world implications. Incorporating novel methodologies such as UV-light mediated sensing enhancement and testing in realistic environments brings us closer to a deployable sensor capable of effectively addressing environmental monitoring and pollution control challenges.

## Supporting Information Available

Schematics of the synthesis of MoS_2_, energy dispersive X-ray spectroscopy (EDX) images of the MoS_2_ device, quantifications of various elements obtained from X-ray photoelectron spectroscopy, transient sensor response at higher concentrations of NO_2_, sensor response to various other gases like NH_3_, NO_2_, CH_4_, and SO_2_, sensor response after 2 months of initial NO_2_ exposure, table of comparison for MoS_2_ sensor response to NO_2_ in the presence of UV light, and simulations of atmospheric chamber for mixing ratios of NO_2_ (PDF)

## Notes

The data sets generated during and/or analyzed during the current study are available in the RADICAL Zenodo repository, https://zenodo.org/communities/radical/
