# Supplementary material for: Exfoliated Molybdenum Disulfide Nanosheet Networks as Sensing Materials for Nitrogen Dioxide Detection
Source: ACS Appl Nano Mater. 2025 Jan 24;8(5):2141–52. doi: 10.1021/acsanm.4c05066 (PMC11811925; doi:10.1021/acsanm.4c05066)
Supplement: Supplementary file 1 — an4c05066_si_001.pdf [file an4c05066_si_001.pdf]

Supporting Information

Exfoliated Molybdenum Disulfide Nanosheet Networks as Sensing Materials for Nitrogen Dioxide Detection

Kusuma Urs MB[1,2], Tian Carey[3,4], Leonidas Tsetseris[5], Shixin Liu[3,4], Kevin Synnatschke[3,4], Zdeněk Sofer[6], Jonathan N. Coleman[3,4], John Charles Wenger[1], Subhajit Biswas[1,2] and Justin D. Holmes[1,2*]*

[1]*School of Chemistry, University College Cork, Cork T12 YN60, Ireland.*

[2]*AMBER Centre, Environmental Research Institute, University College Cork, Cork T23 XE10, Ireland.*

[3]*School of Physics, Trinity College Dublin, Dublin 2, Ireland.*

[4]*Centre for Research on Adaptive Nanostructures and Nanodevices (CRANN) and Advanced Materials Bio-Engineering Research Centre (AMBER), Trinity College Dublin, Dublin2, Ireland.*

[5]*Department of Physics, School of Applied Mathematical and Physical Sciences, National Technical University of Athens, 15780 Athens, Greece.*

[6]*Department of Inorganic Chemistry, University of Chemistry and Technology Prague, Technická 5, Prague 6, 166 28, Czech Republic.*

[*]Corresponding author: s.biswas@ucc.ie or j.holmes@ucc.ie

Figures & Tables

Figure S1 illustrates the schematic representation of the electrochemical exfoliation setup and the exfoliated MoS_2. The MoS_2 flakes were deposited onto a PET substrate featuring gold interdigitated electrodes (IDEs) employed for sensing purposes. Various characterisations were conducted on the exfoliated MoS_2 to analyse its surface morphology.

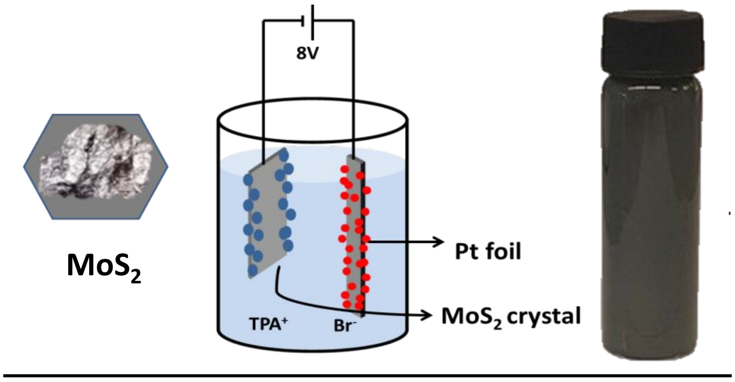

Figure S1: Schematic representation of electrochemical cell used for the exfoliation of MoS_2.

The morphology and surface of the MoS_2 sensor devices were examined on the deposited MoS_2 network prepared using a liquid-phase sonication (LS) method on PET substrates using scanning electron microscopy (SEM), as shown in Figure S2. The SEM images were recorded using a FEI QUANTA 650 field emission scanning electron microscope. Figure S2(a) displays an SEM image of a PET substrate uniformly covered with MoS_2, featuring interdigitated gold electrodes with a finger width and length of 50 μm and 1.1 mm, respectively. The figure illustrates the even distribution of MoS_2 across the substrate, highlighting the effectiveness of the ink jet technique in achieving uniform thin films. Figures S2(b) and S2(c) depict images of the deposited MoS_2 thin film at lower and higher magnifications, respectively. Additionally, EDX mapping indicates atomic weight percentages of 34 % for molybdenum and 65 % for sulfur, indicating sulfur deficiency in the MoS_2 sensor devices.

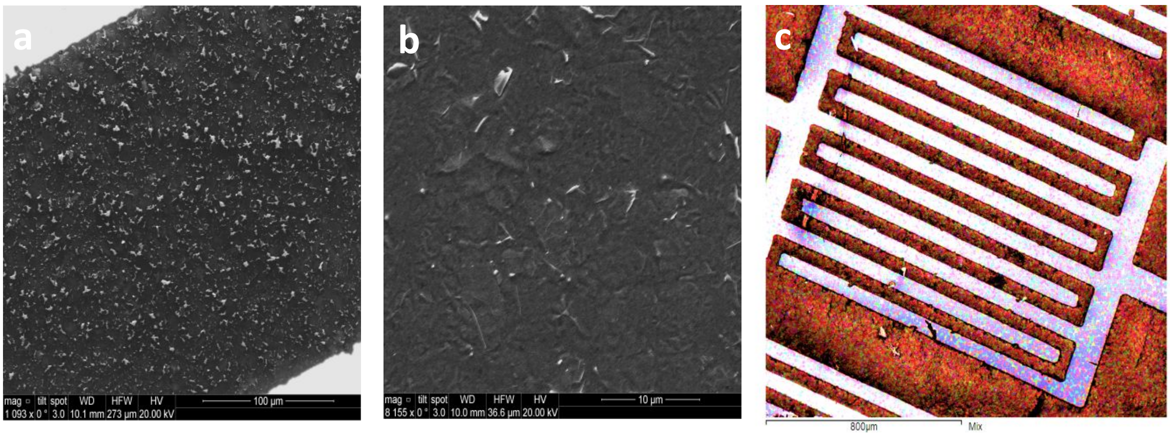

Figure S2: (a), (b) SEM images, and (c) EDX mapping of 2D MoS_2 networks prepared using an LS method on PET.

Table S1: Quantification of various elements in the sensor device using X-ray photoelectron spectroscopy (XPS).

**Name**	**Position**	**FWHM**	**R.S.F.**	**Area**
O 1s	532.4	1.8	2.93	51110
C 1s	284.8	1.6	1	54480
Mo 3d	229.2	0.7	9.5	70874
S 2p	162.0	0.8	1.68	22442
O 1s	532.3	1.9	2.93	50685
C 1s_1	284.8	1.4	1	42535
C 1s_2	285.9	1.4	1	8078
C 1s_3	287.4	0.9	1	836
C 1s_4	288.7	1.4	1	2237
Mo 3d5/2	229.2	0.7	9.5	34366
Mo 3d3/2	232.4	0.9	9.5	24082
S 2p3/2(S^{2-})	162.0	0.7	1.68	12605
S 2p1/2(S^{2-})	163.2	0.7	1.68	6302
S 2p3/2(S_2^{2-})	162.7	1.0	1.68	2439
S 2p1/2(S_2^{2-})	163.9	0.8	1.68	1219

In the XPS analysis, FWHM represents the full width half maxima of the spectra, which measures the equipment's energy resolution. RSF stands for the relative sensitivity factor, which accounts for the efficiency of detecting emitted electrons from different elements.

Optical images were obtained using an Olympus DSX1000 digital microscope, employing a 50× objective lens in bright field mode. Figure S3 displays the optical images of a MoS_2 film deposited on a PET substrate with gold-interdigitated electrodes. The photo reveals MoS_2 flakes typically measuring 2-5 μm in width.

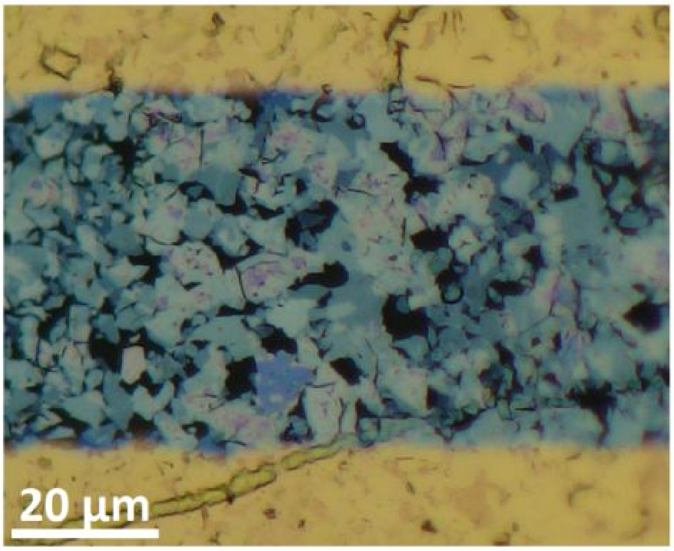

Figure S3: The bright field images of a 2D network of MoS_2 prepared using the LS method on a PET substrate.

The sensor response to higher concentrations of NO_2 ranging from 5-50 ppm was measured at a constant bias voltage of 1 V, as shown in Figure S4. The sensor shows a faster response time of approximately ~2 minutes at higher concentrations. Additionally, the saturation of the sensor current is observed at higher concentrations, indicating complete surface coverage with the analyte molecules.

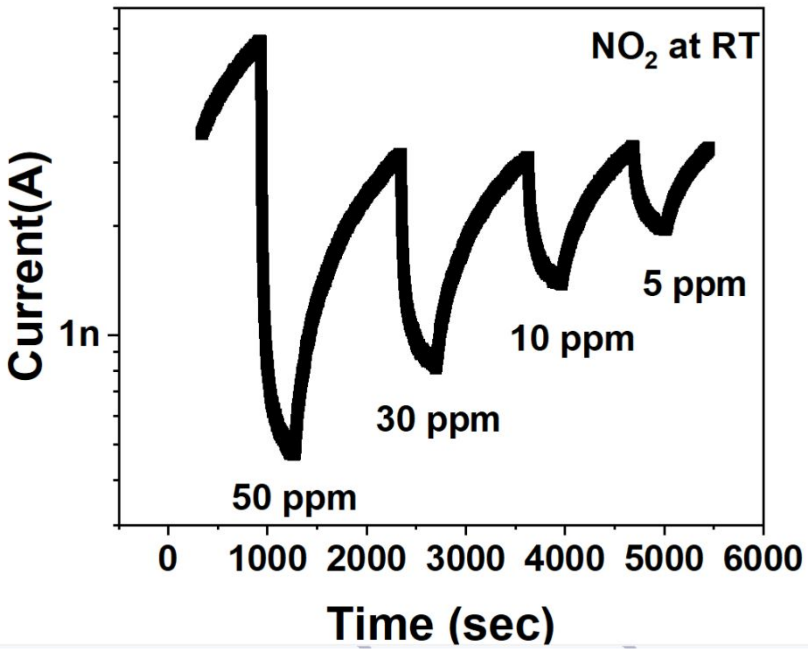

Figure S4: Transient sensor response for NO_2 at room temperature concentrations ranging from 5 to 50 ppm.

Sensor Response in Other Gases: Figure S5(a) shows the response curve for 1 ppm of NH_3. The sensor did not recover, even partially, after removing the gas. The response to a mixture of NO_2 and NH_3 is represented in Figure S5(b). The initial spike seen in Figure S5(b) is due to the time delay in adjusting the two flowmeters. Furthermore, no significant response was detected to 1 ppm of CH_4 and SO_2 individually, as shown in Figure S5(c). The same figure also shows the response to a mixture of NO_2 with SO_2 and CH_4. Finally, the mix of all gases showed a decrease in current, indicating a dominant response to NO_2, as illustrated in Figure S5(d).

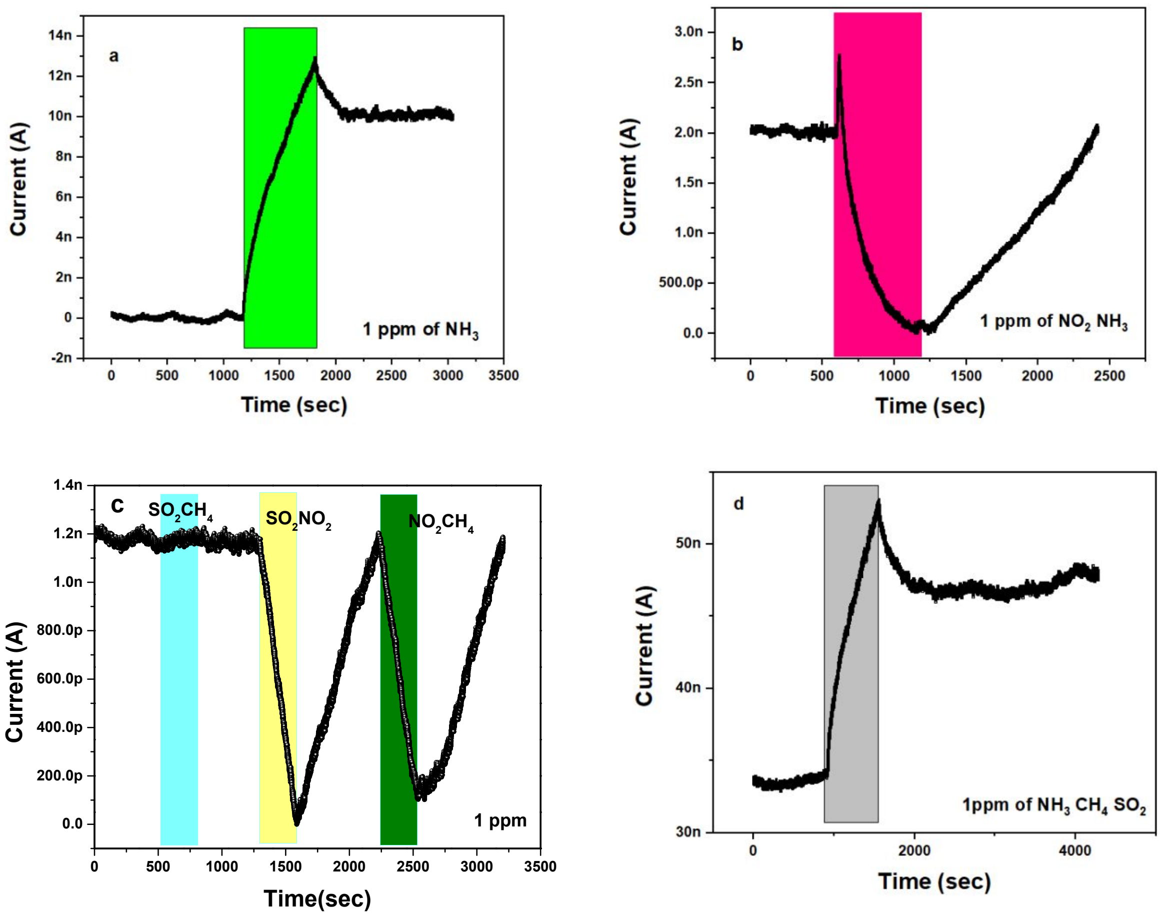

Figure S5: Cross sensitivity measurements of a sensor with 1 ppm each of (a) NH_3, (b) NO_2 and NH_3, (c) a binary mixture of SO_2 and CH_4, SO_2 and NO_2 and SO_2 and CH_4 and (d) a ternary mixture of NO_2, NH_3, CH_4 and SO_2.

Sensor Stability: The transient sensor response for NO_2 gas was measured after two months to assess its stability, as shown in Figure S6. The response for 100 ppb is around 41 %, while for 1 ppm is around 95 %. Although the response for 1 ppm remains consistent with previous measurements, the response for 100 ppb decreased, but not by more than 10 % of the original response. Hence, this demonstrates that the devices maintain stability over an extended period.

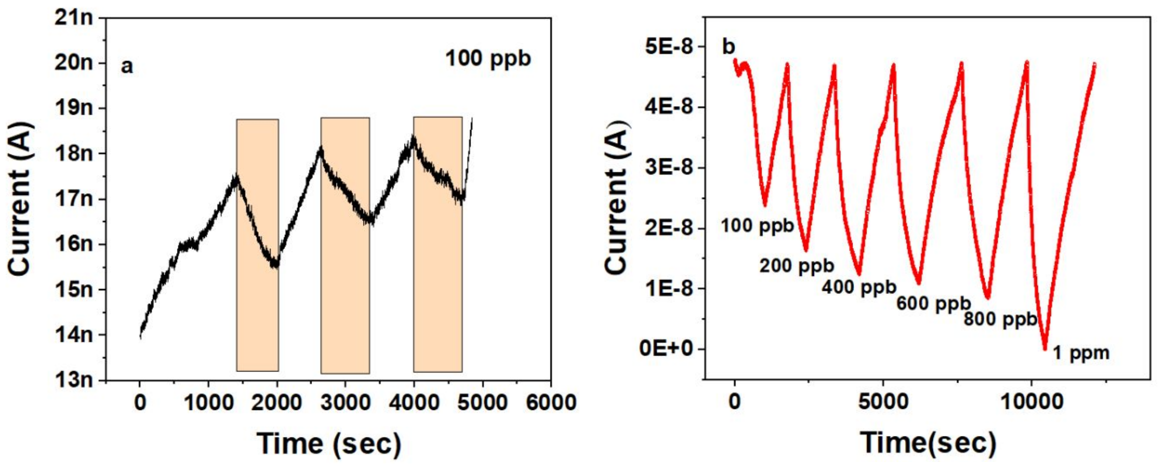

Figure S6: (a) Sensor response for 100 ppb of NO_2 and (b) transient response of a MoS_2 sensor to NO_2 after two months following the initial exposure to NO_2 gas.

Table S2: Comparison of NO_2 sensing responses of MoS_2 prepared using various methods in the presence of UV light

**Material and the substrate**	**Temperature (^oC)**	**Concentration (ppm)**	**Response (%)**	**Response time**	**Recovery time**	**LoD**	**Reference**
Au decorated MoS_2 particles	RT	2.5	30	4 min	14 min	2.5 ppm	[1]
CVD grown MoS_2	RT	100	30	29 s	350 s	5 ppm	[2]
Monolayer MoS_2 on PET (3 terminal)	RT	0.4	670	16 s	65 s	0.4	[3]
Mixed MoS_2 - flakes	RT	10	21.78	6 s	146 s	10 ppm	[4]
MoS_2 nanosheets sensitised with quantum dots	RT	10	615	15 s	62 s	-	[5]
Langmuir Schaefer MoS_2 network on PET	RT(UV)	1	96	300 s	60 s	100 ppb	This work

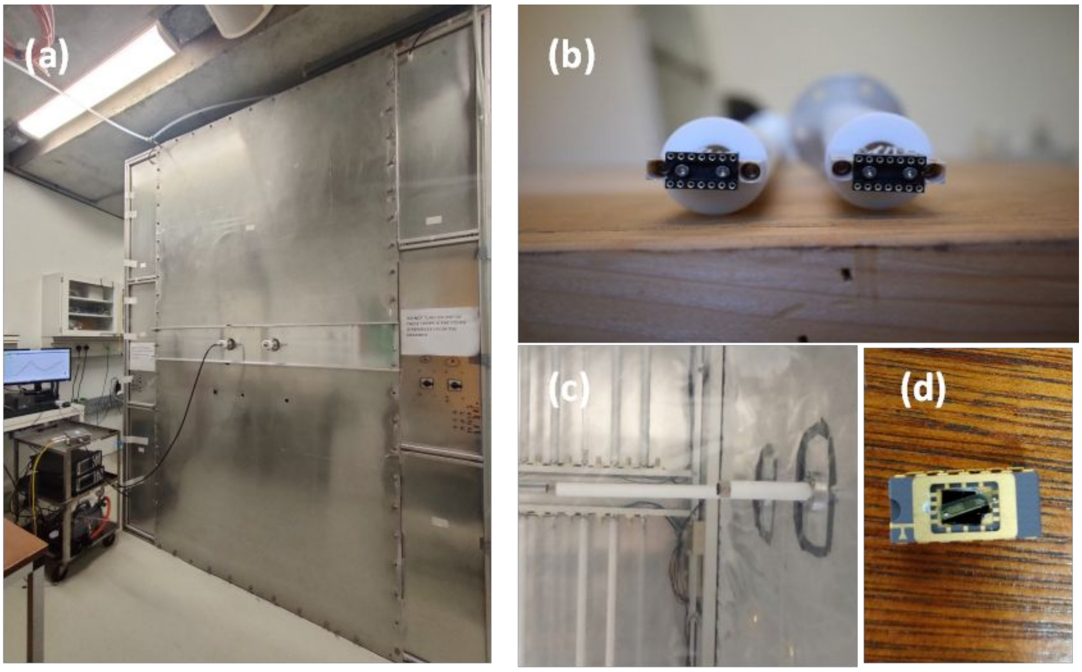

Figure S7: (a) The connection from the sensor to the source metre units (SMUs) within the atmospheric chamber, (b) the cylindrical Teflon arms for the sensor chip, (c) the inside of the chamber fitted with lights and (d) a MoS_2 sensor device bonded onto a chip.

Atmospheric chamber characterisation

To evaluate the mixing ratio of NO_2 within the chamber, a fixed concentration of 1 ppm of NO_2 was introduced into the chamber with varying injection times of 1, 2, 4 and 10 minutes, and the resulting gas concentrations were measured via a NO_2 monitor. The mixing ratio for the chamber used is given in Figure S9. It is noted that a minimum mixing time of 1 hour was needed to achieve the desired concentration of NO_2 within the chamber. Given that the performance of the MoS_2 sensor was evaluated at an optimal concentration of 1 ppm of NO_2 in the probe station, the sensor response for NO_2 within the atmospheric chamber was observed for the same concentration.

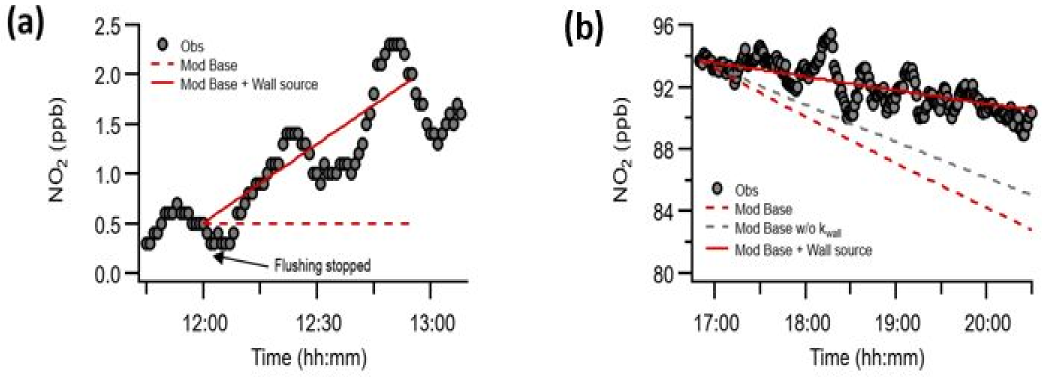

Figure S8: (a) NO_2 time series during a chamber background measurement showing evidence of a NO_2 wall source and (b) NO_2 time series during a chamber kinetics measurement showing evidence of a NO_2 wall source

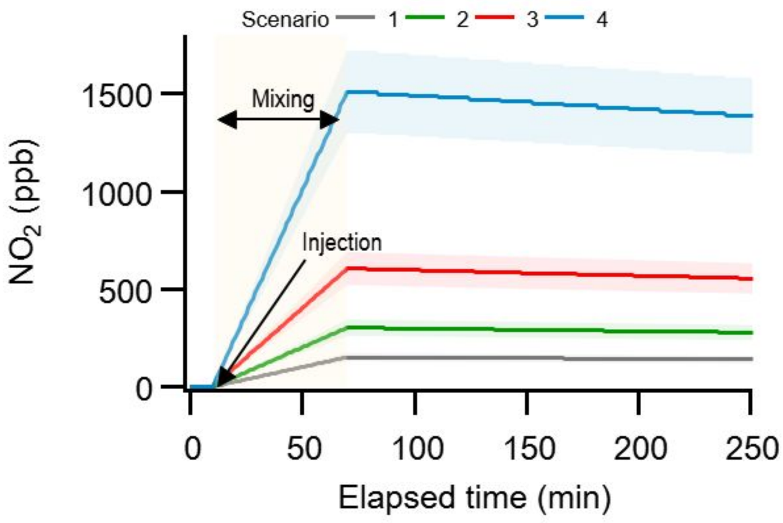

Figure S9: Mixing ratio and the decay of NO_2 gas in the chamber for various concentrations. Scenarios 1, 2, 3, and 4 relate to injections of NO_2 for 1, 2, 4 and 10 minutes.
